# The absorptive effects of orobuccal non-liposomal nano-sized glutathione on blood glutathione parameters in healthy individuals: A pilot study

**DOI:** 10.1371/journal.pone.0215815

**Published:** 2019-04-30

**Authors:** Brianna K. Bruggeman, Katharine E. Storo, Haley M. Fair, Andrew J. Wommack, Colin R. Carriker, James M. Smoliga

**Affiliations:** 1 Department of Chemistry, High Point University, High Point, North Carolina, United States of America; 2 Department of Biology, High Point University, High Point, North Carolina, United States of America; 3 Department of Exercise Science, High Point University, High Point, North Carolina, United States of America; 4 Department of Physical Therapy, High Point University, High Point, North Carolina, United States of America; Tufts University, UNITED STATES

## Abstract

**Background:**

Glutathione is an endogenous antioxidant found in oxidized (GSSG) and reduced (GSH) forms. Glutathione depletion is indicative of oxidative stress and occurs in various pathological conditions and following extreme exercise activity. Raising blood glutathione concentration has potential to attenuate and prevent chronic disease and also to improve recovery from exercise. There are a number of challenges to achieving this through traditional dietary supplements, and thus there is a need to develop optimized delivery methods to improve blood glutathione status. This study evaluated the effect of a novel glutathione formulation on blood glutathione parameters in healthy individuals.

**Methods:**

15 (8 male) healthy individuals (25±5y old, 78.0±14.6kg) participated in a single-blinded randomized placebo-controlled crossover study, with a minimum one-week washout period between treatments. Participants were overnight fasted and administered 1mL of a non-liposomal nano-size glutathione solution (NLNG) containing 200mg of glutathione or 1mL of placebo lacking glutathione. The solution was held in the mouth for 90 seconds before the remainder was swallowed. Blood was collected at baseline, 5, 10, 30, 60 and 120 minutes post-treatment. Protein-bound plasma and erythrocyte lysate concentrations of GSH and GSSG were measured at all time points using previously validated procedures. Linear mixed effects models were used to compare differences between baseline and post-treatment glutathione concentrations between NLNG and placebo for each parameter.

**Results:**

There was a significant main effect for treatment type, such that increases in GSH concentration in erythrocyte lysate were greater following NLNG than placebo (p = 0.001). Similar significant main effects for treatment were also found for total (protein bound + erythrocyte lysate) GSH (p = 0.015) and GSSG (p = 0.037) concentration, as well as total blood glutathione pool (GSH+GSSG, p = 0.006).

**Discussion:**

NLNG increased multiple blood glutathione parameters compared to placebo. Future research should examine whether NLNG can attenuate oxidative stress.

## Introduction

Glutathione is an endogenous antioxidant which is involved in numerous signaling pathways throughout various tissues in the body. Oxidized glutathione (GSSG) can be reduced by glutathione reductase [1]. In its reduced state (GSH), glutathione serves to mediate oxidative stress and signaling by covalently scavenging various reactive species, such as hydrogen peroxide and superoxide, as a ubiquitous low-molecular-weight thiol [1]. Glutathione synthesis can occur in most cells types, with the liver having the greatest role in synthesis and interorgan homeostasis [2], and thus it is not an essential nutrient. Nonetheless, GSH depletion can occur in various conditions associated with excessive formation of reactive oxygen and nitrogen species (RONS) [3]. This excessive production of radical oxidants can occur in pathological states where mitochondrial function is impaired [3], as is common in numerous metabolic diseases [4–6], as well as infection.

Blood glutathione concentration is reported to be decreased in various patient populations compared to healthy controls [7]. For instance, GSH is decreased in the erythrocytes, monocytes, and plasma of individuals with type II diabetes mellitus, compared to healthy individuals [8]. Select studies have also reported high blood glutathione levels to be associated with greater health, especially in elderly individuals [9]. In addition to disease states, extreme exercise causes nitro-oxidative stress and can disrupt resting glutathione levels. For example, a phenomenon known as “glutathione depletion,” in which GSH concentration has decreased substantially below baseline levels, has been observed to persist for 28 days following a 233 km running event [10]. Likewise, well-trained endurance skiers experience a significant decrease in total glutathione levels within erythrocytes 18 hours after competition [11].

The association between glutathione levels and health status has generated interest in increasing glutathione in both athletes and non-athletes, and this has been attempted using various dietary supplementation methods [12, 13]. The half-life of GSH in the plasma (*t* = 1.6 min) is known to be quite short [14], which is believed to limit the potential for oral glutathione supplements. Since glutathione is rapidly oxidized, much of the glutathione in a supplement would not be absorbed by the gastrointestinal tract, and thus would not to be especially effective for substantially raising tissue glutathione concentration. It also appears that the long-term magnitude of response to blood glutathione concentration is dose-dependent [15]. Thus, there is some promise in improving blood glutathione status through supplementation [16, 17], however, there is a need to explore ways to optimize this effect. To date, no studies have attempted to alter blood glutathione levels through using novel formulations specifically designed to maximize absorption.

Increasing the absorption of glutathione into the blood may be achieved through novel formulations which increase the likelihood of this water-soluble antioxidant crossing the hydrophobic plasma membrane. In other antioxidant compounds, this has been achieved by using various nanoformulations, which increase stability and solubilization, increase uptake into cells, improve accessibility to otherwise difficult to reach targets, and increase time spent in the circulation [18–21]. Additionally, there is growing interest in utilizing orobuccal absorption to improve bioavailability of various antioxidants and the speed of absorption [22]. As such, the purpose of this pilot study was to evaluate whether a novel formulation of non-liposomal nano-sized glutathione (NLNG) acutely raises blood glutathione concentration following an administration procedure incorporating orobuccal absorption.

## Materials and methods

### Human Participants

This research was approved by the High Point University Institutional Review Board. The protocol number was 201704–604. Written informed consent was obtained from each participant.

Healthy, non-smoking adults between the ages of 18 to 50 years were recruited to participate in this study. This population was intended to be a sample of convenience, representative of the general healthy public. Individuals with a history of any digestive, cardiovascular, or metabolic diseases which would interfere with glutathione absorption or metabolism, knowingly pregnant and nursing females, and individuals taking daily oral prescription medication were excluded from the study to minimize confounding factors which could influence glutathione levels. Individuals with hemophilia or vascular disorders which would make intravenous catheterization difficult, as well as those allergic to almonds (due to the almond oil base of the intervention) were excluded for safety reasons. All subjects underwent written informed consent.

15 of the 16 recruited healthy individuals completed the study. One individual withdrew from the study at the beginning of her first laboratory visit due to technical difficulties with the venous catheter placement. Summary demographic information from the 15 participants that completed the study are found in Table 1.

**Table 1:** Summary of participant characteristics.

Participants	*N*	Age (years)	Height (cm)	Mass (kg)	BMI (kg/m^2^)
Male	8	28 ± 6	179 ± 7	87.6±13.5	27.2±3.6
Female	7	22 ± 1	165 ± 4	67.0±4.6	24.6±1.8
Combined	15	25±5	173±9.3	78.0±14.6	26.0±3.1

### Experimental design

This study was a single-blinded placebo-controlled randomized crossover trial.

Participants visited the Human Biomechanics and Physiology Laboratory on two occasions, separated by at least 1 week. For each occasion, the participant ingested 1 mL of either NLNG or placebo solution, which were supplied by Nanoceutical Solutions Pelame, LLC. The solutions were administered to participants in a randomized order. Blood glutathione concentration was quantified before and after administration of each treatment, as described in detail below.

### Glutathione and placebo solutions

The NLNG solution consisted of pharmaceutical grade reduced L-glutathione (200 mg/mL), almond oil, silica gel, stevia, and orange oil. Particle size was previously quantified as D[3,2] = 370m (Sauter mean), D[4,3] = 567nm (De Brouckere mean), D50 = 449nm (median), and D80 = 777nm. The placebo solution consisted of the same ingredients, except did not contain glutathione.

The orobuccal exposure time for the treatment was selected based upon unpublished benchtop experiments, which revealed >80% of the glutathione formulation diffused across a Franz diffusions cell and membrane assembly within 90 seconds.

The dosage was meant to be within the range of other published studies which have examined glutathione treatment in healthy individuals.

### Data collection

Participants were fasted from everything but water for at least 8 hours prior to each laboratory session, and this was confirmed through verbal inquiry. At the start of each laboratory session, an intravenous catheter was placed in the antecubital vein of the participant. A baseline blood sample was collected prior to any treatments.

Prior to administration of the treatment, participants were provided instructions on the proper process for receiving the respective fluid. The same procedures were used for NLNG and the placebo. The participant was not informed which product he/she received, and was not shown the product, and thus remained blinded to his/her condition during each laboratory visit.

The researcher drew 1 mL of the fluid from the respective bottle using the supplied graduated dropper. The researcher then used this dropper to place all of the fluid underneath the participant’s tongue. A digital stopwatch was used to measure the elapsed time following the administration of the product. At the 90 second time point, the participant was instructed to swallow the remaining fluid. The researcher monitored the participant throughout the process to ensure that the fluid was not swallowed ahead of this time point.

The digital stopwatch was then reset and restarted with timepoint zero corresponding to the moment the solution was swallowed (post-treatment). Blood was collected from the intravenous catheter at 5, 10, 30, 60, and 120 minutes post-treatment. Standard procedures for blood collection and catheter maintenance were used throughout the study. This included removing approximately 2 mL of blood, which was then discarded, followed by removal of approximately 5 mL of blood to be used for analysis. Following each blood collection procedure, the catheter was flushed with a prepackaged saline syringe. The catheter was removed after the 120-minute data collection period.

### Glutathione quantification from protein-bound glutathione and erythrocyte lysate

The protocol utilized was the enzyme recycling assay described by Rahman *et al*. [23]. Total GSH, GSSG, and GSH was quantified from plasma protein-bound glutathione, erythrocyte lysate, and free plasma.

De-identified whole blood from each time point was immediately divided in two 2-mL portions in EDTA-treated tubes and placed at 4°C. One 2-mL portion was dedicated for analysis of GSH, GSSG, and total GSH from erythrocyte lysate and the other 2-mL portion was used to determine protein-bound glutathione. For analysis of protein-bound glutathione, the whole blood was spun at 1000*g* for 10 min at 4°C. The plasma supernatant was transferred and the remaining cell pellet was diluted with 2.6 mL of 0.1% Triton X-100 and 0.6% sulfosalicylic acid in KPE (0.1 M potassium phosphate buffer with 5 mM EDTA disodium salt, pH 7.5) for homogenization. To the viscous solution was added 1 mL of 1% NaBH_4_ in KPE buffer for a 15-min reaction period at 22°C. The reaction was quenched using 0.4 mL 30% metaphosphoric acid in KPE, followed by centrifugation at 4°C, 1000*g* for 15 min. The supernatant was then used in the GSH, GSSG, and total GSH assays.

Sample preparation for erythrocyte lysate analysis commenced by centrifugation of the 2-mL portion at 4°C, 2500*g* for 5 min. The supernatant was removed and 4 mL of 5% metaphosphoric acid KPE solution was added for vigorous pellet re-suspension. The resultant solution was then spun at 3000*g* at 4°C for 10 minutes and the clear supernatant was collected for GSH, GSSG, and total GSH analysis.

The 96-well microplate reader was programmed to measure absorbance at 412 nm every 30 sec for 5 min. Reagent preparation was as follows: [5,5'-dithio-bis(2-nitrobenzoic acid)] (DTNB) was prepared as a 0.66 mg mL^–1^ solution in KPE, 40 μL of a 250 units mL^–1^ glutathione reductase (GR) stock was diluted with 3 mL KPE, and β-NADPH was also prepared as a 0.66 mg mL^–1^ solution in KPE. GSH stock solutions were freshly prepared by dissolving 1 mg GSH mL^–1^ in KPE. This GSH stock solution was diluted 1:100 with KPE to make a working solution of 10 μg mL^–1^. Further dilution afforded a range of twofold concentrations from 0.103 nM to 26.4 nM. GSSG standards were prepared in a similar fashion to result in a 0.103–26.4 nM concentration series. Stock and sample solutions were kept at 4°C and protected from light during microplate loading.

For analysis of total GSH, the aforementioned erythrocyte lysate or plasma protein-bound glutathione solutions were added to the microplate wells in 20 μL volumes followed by immediate 120 μL addition of a freshly mixed solution of 1:1 GR and DTNB. Following a 30-sec incubation, 60 μL of β-NADPH was added and microplate reader data acquisition was performed. The analysis was performed in triplicate and repeated at least twice. Using the GSH and GSSG standards, the reaction rate (change in absorbance min^–1^) was plotted versus a concentration range of 0.165 nM to 1.65 nM GSH or GSSG to construct calibration curves.

For analysis of GSSG, a 100 μL aliquot of erythrocyte lysate or plasma protein-bound glutathione solution was treated with 2 μL of 2-vinylpyridine as a 10% solution in KPE (*v/v*) at 22°C for 1 h in a well-ventilated fume hood. Following this incubation, 6 μL of triethanolamine was added as a 20% solution in KPE (*v/v*) for vigorous mixing. The solution was placed back at 4°C and microplate reader data was acquired as described above.

Intra- and inter-assay coefficient of variation (CV) values for erythrocyte lysate and protein-bound GSH data sets were calculated in-house to support repeatability of the enzymatic recycling assay described by Rahman et al [23]. Intra-assay CV values were calculated from data collected in triplicate and are found in the supplementary materials (S1 Table). Using the intra-assay CV values, the inter-assay CV values were 5.79 ± 3.35% and 5.89 ± 1.77% for erythrocyte lysate and protein-bound GSH, respectively.

### Data reduction

Means, standard deviations, and coefficients of variation were computed using the triplicate samples from each time point for each treatment. For any data point in which the coefficient of variation was >20%, the anomalous data point was suspected to be erroneous and was removed from the analysis.

Data inspection revealed that free plasma GSH and free plasma GSSG minimally contributed to the total GSH, total GSSG, and total GSH+GSSG (<1%). Because of this minimal contribution and large coefficient of variation compared to plasma protein-bound glutathione and erythrocyte lysate, free plasma glutathione levels were not analyzed in further detail. Thus, total GSH (GSH_Total_) was computed as the sum of erythrocyte lysate GSH (GSH_Lysate_) and protein-bound GSH (GSH_Protein_). The same computations were made for GSSG (GSSG_Lysate_ + GSSG_Protein_ = GSSG_Total_). Total blood glutathione pool (GSH+GSSG_Total_) consisted of the sum of lysate GSH+GSSG (GSH+GSSG_Lysate_) and protein-bound GSH+GSSG (GSH+GSSG_Protein_). The ratio of reduced to oxidized glutathione was computed as GSH/GSSG for lystate (GSH/GSSG_Lysate_), protein (GSH/GSSG_Protein_), and total glutathione pool (GSH/GSSG_Total_).

Analysis of GSSG_Protein_ data from one visit of one participant (#15) were not available due to a technical issue. Therefore, GSSG_Total_ and GSH+GSSG_Total_ were not measured for this participant. All other erythrocyte lysate and protein-bound GSH and GSSG were sufficient for analysis. Baseline value of GSH/GSSG_Lysate_ for one subject was found to be extreme, and that data point was removed from analysis for the corresponding baseline parameter.

The absolute difference in concentration between baseline value and each time point was computed for each glutathione parameter.

### Statistical analysis and interpretation

All statistical analyses were performed in IBM SPSS v24.0. Statistical significance was set at p<0.05 *a priori*.

Linear mixed effects model analyses were performed for each glutathione parameter (erythrocyte lysate, protein-bound, and total for GSH, GSSG, GSH+GSSG, and GSH/GSSG concentrations, a total of twelve parameters). Linear mixed effects models were developed according to recommended practices for repeated-measures data. Diagonal, scaled-identity, autoregressive (standard, heterogeneous, and moving average variations), and compound symmetry repeated measures covariance structures were all tested, and the model with the lowest Akaike’s Information Criteria was considered to be the best fit. If the best fitting model did not have significant two-way interactions (i.e., 1) time point x treatment type, 2) time point x baseline value covariate, 3) treatment type x baseline value covariate) or a three-way interaction (i.e., time point x treatment type x baseline value covariate), the non-significant interactions were removed from the model and only the main effects were compared.

Separate statistical analyses were used to: 1) compare baseline values between treatment groups, 2) compare post-treatment treatment differences in concentration for each glutathione parameter from respective baseline values across all time points, and 3) compare maximum post-treatment change from baseline for each glutathione parameter.

The rationale behind these separate analyses was that a comparison of post-treatment differences from baseline between the two different treatments was the primary interest. Thus, including baseline values as a distinct time point in the latter model would complicate interpretation if a significant main effect was identified. Nonetheless, baseline value was included as a covariate to ensure that any influence of baseline value on post-treatment effect was still included in the analysis.

### Baseline differences and test sequence

Because this study utilized a crossover design, the statistical analyses of baseline scores should include analyses to determine a sequence effect was present. In other words, data were analyzed to determine if baseline values were dependent upon the sequence of treatments (NLNG-then-placebo versus placebo-then-NLNG).

Baseline value was the dependent variable. Treatment type (NLNG vs. placebo) served as a repeated measures categorical independent predictor variable. Additionally, the sequence of treatment served as an independent predictor. The two-way interaction between treatment type and sequence of testing was included in the model. Participant was included as a random effect.

### Post-treatment changes

For each glutathione parameter, difference in concentration from respective baseline value was the dependent variable. Time point (i.e., 5, 10, 30, 60 and 120 minutes post-treatment) and treatment type (NLNG vs. placebo) served as repeated measures categorical independent predictor variables. Baseline concentration served as a covariate. Additionally, the two-way interactions and three-way interaction between time point, treatment, and baseline value was included in the model. Participant was included as a random effect.

For each participant, the time point at which the maximum change in each glutathione parameter occurred was determined (analogous to C_max_ in pharmacokinetic literature). The median of these values was then computed to determine the time point at which maximum change occurred (analogous to T_max_ in pharmacokinetic literature).

### Interpretation

A significant main effect for treatment type indicated that there was an overall increase in concentration (compared to baseline) for a given glutathione parameter between NLNG and placebo, using pooled across all post-treatment time points. Detailed information regarding interpretation of baseline covariates, two-way interactions, and three-way interactions is found as a supplementary file to this paper (S1 Appendix).

## Results

### Baseline values and sequence of testing

Baseline values for each glutathione parameter are presented in Table 2.

**Table 2:** Summary of baseline values for each glutathione parameter. Data are presented as Mean (Standard Error) [95% confidence interval], based on estimated marginal means from the linear mixed effects model. Identical standard errors for some parameters are due to the statistical models employed assuming homogeneity of variance for both treatment groups (depending on the repeated measures covariance structure employed in the best fitting linear mixed effects model).

		Placebo (μM)	NLNG (μM)	p-value
**GSH**	**Erythrocyte Lysate**	188.1 (20.8)	183.5 (20.8)	0.791
		[144.5, 231.7]	[139.9, 227.1]	
	**Protein-Bound**	535.2 (73.7)	411.2 (73.7)	0.134
		[382.4, 688.0]	[258.3, 564.0]	
	**Total**	723.3 (87.3)	594.7 (87.3)	0.170
		[541.8, 904.8]	[413.2, 776.2]	
**GSSG**	**Erythrocyte Lysate**	61.0 (16.4)	54.4 (16.4)	0.528
		[26.3, 95.8]	[19.7, 89.2]	
	**Protein-Bound**	178.2 (62.3)	142.1 (62.3)	0.133
		[43.3, 313.0]	[7.2, 277.0]	
	**Total**	240.4 (77.4)	196.7 (77.4)	0.095
		[72.7, 408.2]	[29.0, 364.5]	
**GSH+GSSG**	**Erythrocyte Lysate**	243.7 (26.0)	232.5 (35.9)	0.603
		[187.6, 299.9]	[154.4, 310.7]	
	**Protein-Bound**	724.4 (124.7)	555.5 (124.7)	0.074
		[459.0, 989.8]	[290.1, 820.9]	
	**Total**	969.3 (151.2)	782.2 (151.2)	0.078
		[646.6, 1292.0]	[459.5, 1104.9]	
**GSH/GSSG**	**Erythrocyte Lysate**	6.8 (2.1)	5.4 (0.8)	0.268
		[2.2, 11.5]	[3.6, 7.2]	
	**Protein-Bound**	6.6 (2.1)	4.6 (0.7)	0.290
		[2.1, 11.0]	[3.2, 6.0]	
	**Total**	6.4 (1.8)	4.2 (0.5)	0.259
		[2.6, 10.2]	[3.1, 5.3]	

There were no statistically significant two-way interactions for treatment type and testing sequence. Baseline values were not significantly different between treatment types. These data verified that the washout period was sufficient.

### Post-treatment changes from baseline

**Fig 1:**
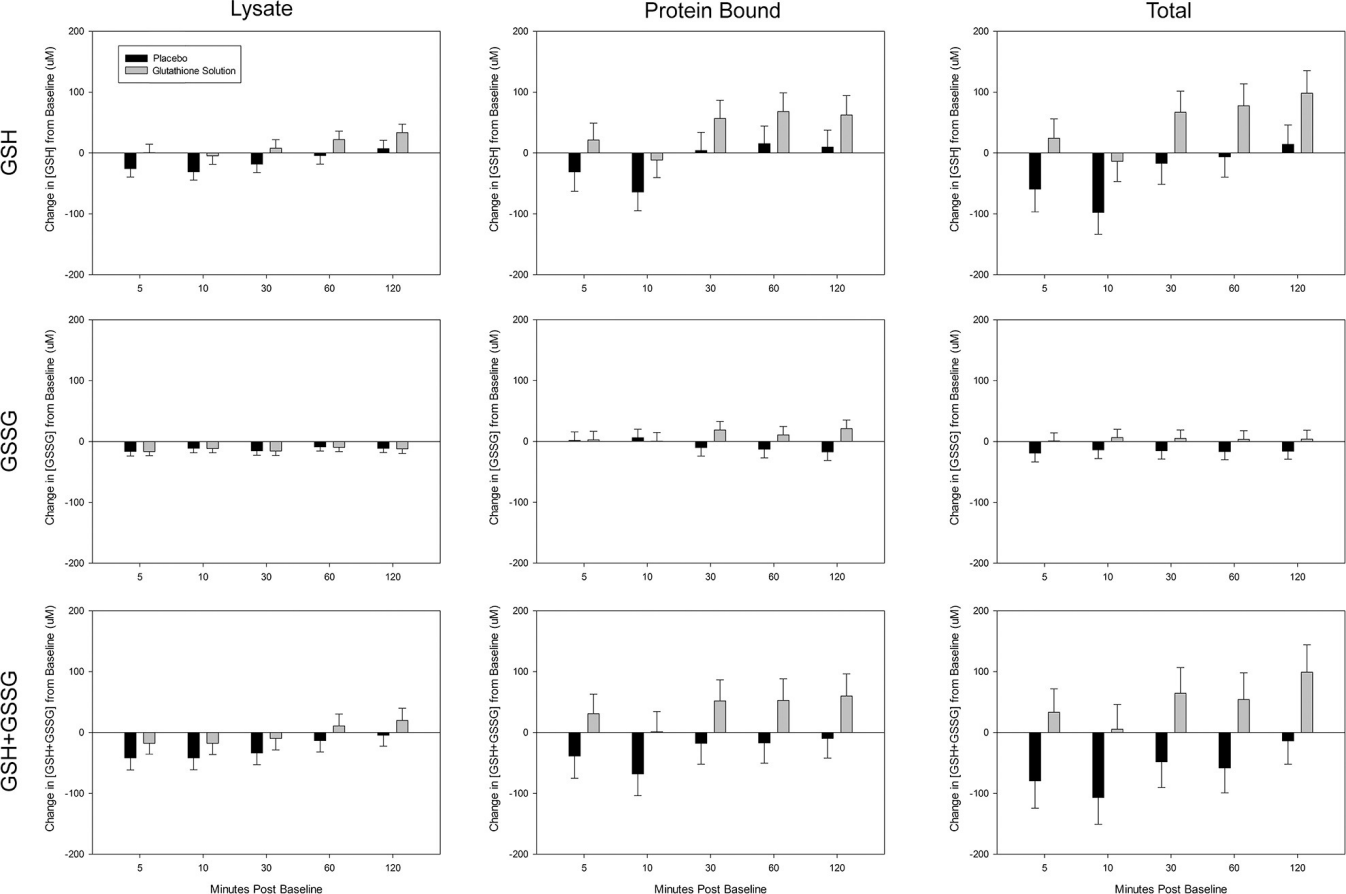
Changes in glutathione parameters across all five post-treatment time points following administration of placebo and NLNG. There was a main effect for NLNG to increase GSH_Lysate_, GSH_Total_, GSSG_Total_, and all GSH+GSSG parameters. This treatment specific increase was not specific to any one post-treatment time point. Greater detail regarding statistical significance is presented in the results of the main text and the online supplement (S1 Appendix).

Fig 1 summarizes the change from baseline for each glutathione parameter for NLNG and placebo. As described below, NLNG increased multiple glutathione parameters (as demonstrated by a significant main effect for treatment type), but did not do this at any one-specific post-treatment time point (as demonstrated by a lack of significant two-way interaction for treatment type x time). The detailed results from the comprehensive statistical analysis are found as supplementary material to this paper (S1 Appendix).

#### GSH

GSH_Lysate_ (p = 0.001) and GSH_Total_ (p = 0.015) were significantly increased from baseline following NLNG compared to placebo. However, the change in GSH_Protein_ did not differ from baseline between NLNG and placebo (p = 0.061).

#### GSSG

GSSG_Total_ was significantly greater following NLNG than placebo (p = 0.004). Treatment type did not influence the change from baseline in GSSG_Lysate_ (p = 0.936) and GSSG_Protein_ (p = 0.054).

#### GSH+GSSG

The change from baseline for GSH+GSSG_Protein_ (p = 0.034) and GSH+GSSG_Total_ (p = 0.006) were greater following NLNG than the placebo.

#### GSH/GSSG

The change from baseline for GSH/GSSG_Lysate_ was significantly greater for NLNG than placebo (p = 0.045). Treatment type did not influence the change from baseline in GSH/GSSG_Protein_ (p = 0.276) or GSH/GSSG_Total_ (p = 0.216).

#### Maximum change from baseline

Table 3 presents the maximum changes from baseline for GSH, GSSG, and GSH+GSSG parameters.

**Table 3:** Maximum change from baseline in GSH parameters. GSH_Lysate_, GSH_Total_, and GSH+GSSG_Total_ were significantly increased compared following NLNG compared to placebo.

		Placebo (μM)	NLNG (μM)	p-value
**GSH**	**Erythrocyte Lysate**	21.6 (7.3)	59.5 (7.3)	<0.001
		[7.2, 36.1]	[45.1, 73.9]	
	**Protein-Bound**	73.8 (34.7)	142.2 (34.7)	0.175
		[2.7, 144.9]	[71.1, 213.3]	
	**Total**	50.6 (39.8)	184.5 (39.8)	0.025
		[-31.0, 132.2]	[102.9, 266.1]	
**GSSG**	**Erythrocyte Lysate**	14.5 (6.8)	10.2 (11.2)	0.708
		[0.0, 29.1]	[-13.8, 34.3]	
	**Protein-Bound**	28.6 (16.3)	66.8 (16.3)	0.110
		[-4.9, 62.2]	[33.2, 100.3]	
	**Total**	21.2 (16.6)	67.7 (21.8)	0.102
		[-14.6, 57.0]	[20.7, 114.7]	
**GSH+GSSG**	**Erythrocyte Lysate**	26.4 (21.5)	61.3 (21.5)	0.260
		[-17.6, 70.4]	[17.3, 105.3]	
	**Protein-Bound**	63.2 (39.8)	164.5 (39.7)	0.153
		[-19.1, 145.6]	[82.1, 246.9]	
	**Total**	25.4 (44.1)	205.6 (44.1)	0.035
		[-66.3, 117.1]	[113.9, 297.3]	

#### Median time to maximum change from baseline

For all nine glutathione concentration parameters, the median time point at which maximum change took place was 30 minutes.

## Discussion

This study demonstrated that NLNG acutely increased total blood glutathione concentration using proven spectrophotometric detection methods to quantify reactive thiol species. While the analytical protocol does not disambiguate the structure of the reactive thiol species, direct reaction of GSH or other GSH-derived compounds, such as γGlu-Cys and Cys-Gly, is reliably determined using this methodology [23]. Fig 1 suggests that this increase in total glutathione pool appears to mostly result from increased GSH bound to plasma proteins, though GSH in erythrocyte lysate and GSSG bound to plasma proteins also contribute to this. The baseline values for glutathione parameters in this study are generally within the range reported in other studies [24, 25]. However, changes in GSH parameters within this study are not readily comparable to that of other studies attempting to alter blood glutathione pool due to differences in research methodology, including the specialized nanoformulation of glutathione utilized in this study.

The primary goal was simply to determine if NLNG administration could alter glutathione parameters, and if so, which parameters were altered (e.g., oxidized versus reduced, erythrocyte lysate vs. protein bound). The rationale for measuring glutathione levels across multiple time points, rather than a single time point, is that inter-individual variation in absorption is common across various supplements and pharmacological products, and data from a single time point may not be an accurate representative of ability to increase glutathione pool. While area under the curve (AUC) could potentially useful for determining total glutathione absorption into the bloodstream, the relative dearth of literature on glutathione supplementation does not allow for confident interpretation of this type of analysis. For instance, after maximal plasma concentration is reached, it is possible that decreases in blood glutathione parameters could be representative of excretion from the body (e.g., into the urine), metabolism into other compounds, or distribution from the blood into other tissues. Likewise, the mechanism behind the apparent decreases in glutathione following placebo seen in Fig 1 remain uncertain (though a similar trend has been seen in other studies [25]). Though data were collected across multiple post-treatment time points, we caution against using this data to evaluate the pharmacokinetic properties of NLNG, since pharmacokinetic evaluation (e.g., specific details of the time-course of absorption, distribution to tissues besides erythrocytes, and excretion) of NLNG was not a primary goal of this study. Likewise, the duration of increased total glutathione pool following NLNG is not known, as many glutathione parameters appeared to remain elevated at the last post-treatment time point measured (Fig 1).

Although visual inspection of mean data may appear to demonstrate a slight decrease at the 10-minute post-treatment point and increases at the other four post-treatment time points, it is important to recognize that there was not a statistical interaction between treatment type and time. In other words, GSH_Total_ increased in NLNG compared to placebo across all time points (p = 0.015), but from a statistical standpoint, this increase did not differ between individual time points following the treatment. Inter-individual variation in glutathione absorption may be one key reason why there were no statistical differences in [GSH_Total_] between time points following ingestion of the glutathione supplement. Because the time at which GSH_Total_ peaked was inconsistent between individuals, an especially large difference at one specific post-treatment time point would not be expected. Thus, it is not surprising that only a more general main effect for treatment type was observed without a two-way interaction between treatment type and time. Thus, there was value in examining the maximal change from baseline for all glutathione parameters (Table 3). Statistical analysis revealed significant increases in GSH_Lysate_ and GSH_Total_, and ultimately a significant increase in GSH+GSSG_Total_ following NLNG. While the maximal change in GSH_Protein_ and the GSSG parameters were not significantly different between treatments, it is possible that NLNG caused smaller or less consistent increases in these parameters, which cumulatively also contributed to the significant increase in GSH+GSSG_Total_.

**Fig 2:**
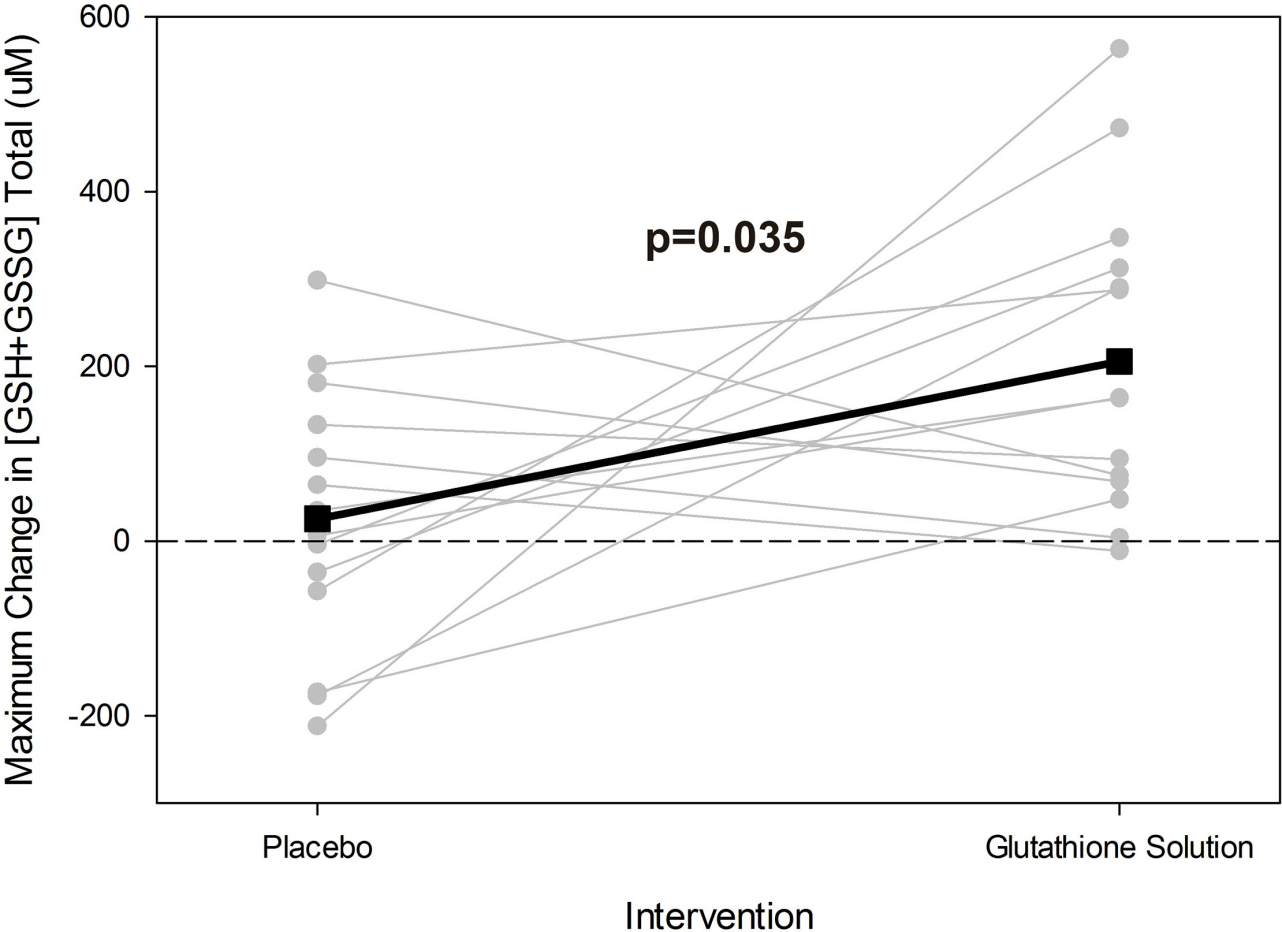
Individual participant data for maximum change in GSH+GSSG_Total_. Most participants had a greater maximum change from baseline values following NLNG compared to placebo. Grey lines represent each individual participant, and the black line represents the mean values. Individual results for this parameter is representative of that from other glutathione parameters.

From a clinical perspective, interventions aimed at increasing the blood glutathione pool do not necessarily need to be rapid, and thus the difference between achieving maximal absorption in minutes versus hours was not as important as the more general ability to simply absorb gluathione into the bloodstream. Given the individuality of the responses to the glutathione supplement, in both timing and magnitude, we also visually compared how the maximum change from baseline value differed between treatment types. Fig 2 confirms that the maximum change in GSH_Total_ was greater following NLNG compared to placebo in most participants. While the present study suggests that glutathione concentration can be elevated following a single dose, future studies should examine whether chronic administration can result in long-term increases in glutathione levels or any beneficial changes in health-related biomarkers.

### Limitations

This was a pilot study to determine whether this novel glutathione formulation could increase GSH and GSSG in the blood, thus there are some intrinsic limitations to this type of study. Even though blood glutathione concentrations were increased compared to placebo, there was some inter-individual variation in this response, likely due to physiological differences in glutathione absorption and metabolism.

Though glutathione should reduce oxidative stress, we did not measure oxidative stress in this study, as this study was focused on healthy participants, rather than individuals expected to have glutathione depletion. It is inherently difficult to examine health benefits of a product on healthy people [26] (e.g., if biomarkers of oxidative stress are not already elevated, it may be difficult to further reduce these values). Thus, future studies should examine the clinical effects of raising glutathione concentration via NLNG treatment.

### Summary

Clinical research aiming to improve glutathione concentration requires sufficient glutathione absorption and delivery. This study adds to emerging evidence that nanoformulation absorption is effective for quickly raising blood glutathione levels in humans. The delivery strategy used in this study, including a novel nanoformulation and an orobuccal administration, may be effective in improving the magnitude of absorption of other modulators of oxidative stress known to have limited bioavailability, such as resveratrol [22]. The results of this study indicate that a formulation of 200 mg NLNG per mL of an almond oil-based solution is effective in raising GSH and GSH_Total_ levels in whole blood of healthy human participants. Given that this study documented NLNG absorption, future research should focus on dose-dependent and long-term studies effect on oxidative stress markers. Continuing research could also focus on the efficacy of NLNG for clinical conditions associated with GSH deficiency.

## Supporting information

S1 Appendix Detailed statistical methods and results. (DOCX)

S1 Table Coefficient of variation for the assays. (XLSX)

S2 Table Dataset for the study. (XLSX)
