# Supplementary material for: The absorptive effects of orobuccal non-liposomal nano-sized glutathione on blood glutathione parameters in healthy individuals: A pilot study
Source: PLoS One. 2019 Apr 30;14(4):e0215815. doi: 10.1371/journal.pone.0215815 (PMC6490881; doi:10.1371/journal.pone.0215815)
Supplement: S1 Appendix — (DOCX) [file pone.0215815.s001.docx]

**Supporting Methods**

**Statistical Interpretation**

A significant main effect for treatment type indicated that there was an overall increase in concentration (compared to baseline) for a given glutathione parameter between NLNG and placebo, using pooled across all post-treatment time points.

A significant main effect for time indicated that the change in concentration (compared to baseline) for a given glutathione parameter differed across at least two post-treatment time points, regardless of treatment type. If this main effect was present, a *post hoc* comparison between individual time points was performed.

A significant main effect for the baseline covariate indicated that the post-treatment change in a given glutathione parameter was dependent upon baseline value regardless of treatment type (e.g., individuals with lower baseline GSH may have a large post-treatment change in GSH, whereas those with a higher baseline GSH may not experience much if any post-treatment change, regardless of whether they received NLNG or placebo.)

A significant two-way interaction between treatment type and time indicated that the change in concentration for a given glutathione parameter was different across time points, and this differed between NLNG and placebo (regardless of baseline value). A significant two-way interaction between treatment type and baseline indicated that the change in concentration for a given glutathione parameter was different between treatment types, but was dependent upon baseline value (regardless of time point). A significant two-way interaction between time and baseline indicated that the change in concentration over time for a given glutathione parameter was dependent upon baseline value, but independent of treatment type. A significant three-way interaction indicated that the post-treatment values for a glutathione parameter varied with time, differed between treatment type, and were dependent upon baseline values.

**Supporting Results**

**Baseline Values and Sequence of Testing**

There were no statistically significant two-way interactions for treatment type and testing sequence. Baseline values were not significantly different between treatment types. The order of testing sequence did result in different baseline values for GSH_Lysate_ (p=0.003), GSH_Protein_ (p=0.027), GSH_Total_ (p=0.028), and GSH+GSSG_Total_ (p=0.014), but not for any of the other parameters. In other words, those randomized to one sequence of testing had higher baseline values on both visits than those randomized to another sequence of testing. Given the lack of two-way interaction, we do not believe there is a physiological explanation for this, and this sequence effect is due to inter-individual variation.

**Baseline Values and Sequence of Testing**

**GSH**

For GSH_Lysate_, there was a significant main effect for baseline covariate (p=0.005), treatment type (p=0.001) and time (p=0.014). There were no significant interactions. *Post hoc* analyses for the time main effect revealed significant differences between 5 and 120 (p=0.009), 10 and 60 (p=0.034), 10 and 120 (p=0.002), 30 and 120 (p=0.040) minutes.

For GSH_Protein_, there was a significant main effect for time (p=0.023), but not treatment type (p=0.061) or baseline covariate (p=0.107). There were no significant interactions. *Post hoc* analyses for the time main effect revealed significant differences between 10 and 30 (p=0.006), 10 and 60 (p=0.003), and 10 and 120 minutes (p=0.008).

For GSH_Total_, there was a significant main effect for time (p=0.009) and treatment type (p=0.015), but not for baseline covariate (p=0.146). There were no significant interactions. *Post hoc* analyses for the time main effect revealed significant differences between 5 and 120 (p=0.026), 10 and 30 (p=0.004), 10 and 60 (p=0.004), and 10 and 120 (p=0.001) minutes.

**GSSG**

For GSSG_Lysate_, there was a significant main effect for baseline covariate (p<0.001), but not treatment type (p=0.936) or time (p=0.707). There were no significant interactions.

For GSSG_Protein_, there was a significant main effect for baseline covariate (p=0.004), but not treatment type (p=0.054) or time (p=0.133). There was a significant two-way interaction for treatment type x baseline (p<0.001), and for time x baseline (p=0.001), but not for treatment type x time (p=0.145). The three-way interaction was also statistically significant (p=0.022).

For GSSG_Total_, there was a significant main effect for baseline covariate (p=0.004) and treatment type (p=0.037), but not for time (p=0.118). The two-way interaction between treatment type x baseline was significant (p<0.001), as well as time x baseline (p=0.002).

**GSH+GSSG**

For GSH+GSSG_Lysate_, there was a significant main effect for baseline covariate (p=0.010), but not treatment type (p=0.093) or time (p=0.061). There were no significant interactions.

For GSH+GSSG_Protein_, there was a significant main effect for baseline covariate (p=0.013) and treatment type (p=0.034), but not time (p=0.418). There were no significant interactions.

For GSH+GSSG_Total_, there was a significant main effect for baseline covariate (p=0.045) and treatment type (p=0.006), but not time (p=0.165). There were no significant interactions.

**GSH/GSSG**

For GSH/GSSG_Lysate_, there was a significant main effect for baseline covariate (p<0.001), treatment type (p=0.045), and time (p=0.002). There was a significant two-way interaction of time x baseline (p<0.001), but no further significant interactions. Pairwise comparisons revealed significant differences between 5 versus 10 minutes (p=0.016) and 10 versus 30 minutes (p=0.002).

For GSH/GSSG_Protein_, there was a significant main effect for baseline covariate (p<0.001), but not for treatment type (p=0.276) or time (p=0.135). There was a significant two-way interaction of time x baseline (p<0.001), but no further significant interactions.

For GSH/GSSG_Total_, there was a significant main effect for baseline covariate (p<0.001), but not for treatment type (p=0.216) or time (p=0.965). There were no significant interactions.

**Maximum Change from Baseline**

There was a significant two-way interaction between treatment type x baseline values for GSSG_Protein_ (p=0.008) and GSSG_Total_ (p=0.028), but not for any of the other parameters. This indicates that the maximal change could be dependent upon baseline values (i.e., if baseline values for a given parameter were low, maximum change may be different than if baseline values for that parameter were high).
